# Elevated activation of CaMKIIα in the CPEB3-knockout hippocampus impairs a specific form of NMDAR-dependent synaptic depotentiation

**DOI:** 10.3389/fncel.2014.00367

**Published:** 2014-11-03

**Authors:** Wen-Hsuan Huang, Hsu-Wen Chao, Li-Yun Tsai, Ming-Hung Chung, Yi-Shuian Huang

**Affiliations:** ^1^Institute of Biomedical Sciences, Academia SinicaTaipei, Taiwan; ^2^Interdisciplinary Program of Life Sciences, National Tsing Hua UniversityHsinchu, Taiwan

**Keywords:** CaMKII, CPEB, depotentiation, NMDAR, synaptic depression

## Abstract

Cytoplasmic polyadenylation element binding protein 3 (CPEB3) is a sequence-specific RNA-binding protein that confines the strength of glutamatergic synapses by translationally downregulating the expression of multiple plasticity-related proteins (PRPs), including the *N*-methyl-D-aspartate receptor (NMDAR) and the postsynaptic density protein 95 (PSD95). CPEB3 knockout (KO) mice exhibit hippocampus-dependent abnormalities related not only to long-term spatial memory but also to the short-term acquisition and extinction of contextual fear memory. In this study, we identified a specific form of NMDAR-dependent synaptic depotentiation (DPT) that is impaired in the adult CPEB3 KO hippocampus. In parallel, cultured KO neurons also exhibited delayed morphological and biochemical responses under NMDA-induced chemical long-term depression (c-LTD). The c-LTD defects in the KO neurons include elevated activation of calcium/calmodulin-dependent protein kinase II alpha subunit (CaMKIIα), increased Ser831 phosphorylation of GluA1 and slow degradation of PSD95 and GluA1. Because transient pharmacological suppression of CaMKIIα activity during the DPT-initiating phase successfully reversed the LTP in the KO hippocampus, DPT and c-LTD in the two different systems shared common molecular defects due to the absence of CPEB3. Together, our results suggest that CPEB3 deficiency imbalances NMDAR-activated CaMKIIα signaling, which consequently fails to depress synaptic strength under certain stimulation conditions.

## INTRODUCTION

The cytoplasmic polyadenylation element binding protein (CPEB) family of RNA-binding proteins and translational regulators contains four members in vertebrates, that is, CPEB1, CPEB2, CPEB3, and CPEB4, all of which are expressed in the brain (Wu et al., 1998; Theis et al., 2003; Huang et al., 2006; Chen and Huang, 2012). All CPEB proteins have a carboxy-terminal RNA-binding domain (RBD) and an amino-terminal regulatory domain; in the case of CPEB1, the latter domain stimulates polyadenylation-induced translation following phosphorylation by Aurora A (Mendez et al., 2000). CPEBs 2–4 were first identified based on their shared 45% sequence identities with CPEB1 in the RBD; however, these proteins share no significant homology with CPEB1 in the N-terminal domain (Richter and Sonenberg, 2005; Ivshina et al., 2014). Despite the sequence disparities with CPEB1, CPEBs 2–4 also regulate translation, but the mechanisms employed by each CPEB to control protein synthesis might be somewhat different. For example, CPEB1 represses target RNA translation via binding to maskin or neuroguidin to prevent the assembly of the eIF4E–eIF4G initiation complex. Following phosphorylation on Thr171, CPEB1 promotes polyadenylation-induced translation initiation; i.e., the elongated poly(A) tails of RNAs are bound by additional poly(A)-binding proteins (PABPs), which recruit eIF4G to compete with maskin for the binding of eIF4E (see reviews in Richter and Sonenberg, 2005; Ivshina et al., 2014). In contrast, CPEB3 represses translation via the downregulation of the GTPase activity of the elongation factor eEF2 (Chen and Huang, 2012). *N*-methyl-D-aspartate receptor (NMDAR) signaling triggers calpain 2-mediated cleavage of CPEB3, which results in the translation of CPEB3-targeted RNAs (Huang et al., 2006; Chen and Huang, 2012; Wang and Huang, 2012). In this molecular model, CPEB3 is a repressor and ameliorates its repression ability via NMDAR-activated proteolysis. Although CPEB3 neither interacts with the cleavage and polyadenylation specificity factor (CPSF) nor requires the AAUAAA hexanucleotide for translational activation (Huang et al., 2006; Wang and Huang, 2012), it has been reported that the monoubiquitination of CPEB3 by neuralized1 switches CPEB3 from a repressor to an activator that increases the polyadenylation-induced synthesis of the subunits of the α-amino-3-hydroxy-5-methyl-4-isoxazolepropionic acid receptors (i.e., the GluA1 and GluA2 subunits of AMPARs; Pavlopoulos et al., 2011). However, it is unclear whether neuronal activity regulates this modification, in which the lysine residue of CPEB3 is conjugated to ubiquitin and how the monoubiquitinated CPEB3 promotes polyadenylation. CPEB1-controlled translation plays important roles in development, the cell cycle, neuronal plasticity and cellular senesce (see Ivshina et al., 2014 for review), yet the physiological functions of CPEBs 2–4 have only just begun to emerge.

Activity-induced synthesis of plasticity-related proteins (PRPs) sustains long-lasting synaptic changes and is essential for long-term memory (LTM) formation. Decades of studies have demonstrated that the syntheses of PRPs can be regulated through translational control (see reviews in Costa-Mattioli et al., 2009; Richter and Klann, 2009; Darnell and Richter, 2012; Gal-Ben-Ari et al., 2012). Because all CPEBs are expressed in neurons and might regulate translation-dependent synaptic modifications, the roles of CPEBs in learning and memory have been investigated using mice in which individual *cpeb* genes have been genetically ablated. In the hippocampus-dependent Morris water maze and contextual fear conditioning test, mice in which the *cpeb1* gene have been ablated exhibited reduced extinction in spatial and fear LTMs (Berger-Sweeney et al., 2006). Disruption of the *cpeb3* gene enhances the consolidation of spatial LTM but only affects the short-term acquisition and extinction of contextual fear memories (Chao et al., 2013). In contrast, mice without the *cpeb4* gene exhibit no apparent hippocampus-related memory deficits (Tsai et al., 2013). Although the role of CPEB2 in learning and memory has yet to be uncovered, it appears that the absence of different CPEBs differentially affects learning and memory.

We previously identified several molecules at glutamatergic synapses that are translationally up-synthesized in the CPEB3 knockout (KO) neurons and brains including the scaffolding protein postsynaptic density protein 95 (PSD95) and subunits of the NMDARs (i.e., NR1, NR2A, and NR2B) and AMPARs (i.e., GluA1 and GluA2). Moreover, the increased NMDAR expression results in greater NMDA-induced calcium influx in KO neurons (Chao et al., 2013). Nevertheless, several long-lasting forms of synaptic transmission, including the LTP evoked by one train or four trains of high-frequency stimulation (HFS) and theta burst stimulation and the long-term depression (LTD) induced by paired-pulse low HFS (PP-LFS), appear normal in the Schaffer collateral (SC)-CA1 pathway of adult KO hippocampal slices (Chao et al., 2013). To extend the results of the previous study, cultured neurons and hippocampal slices prepared from wild-type (WT) and KO littermates were used to further investigate whether any other activity-regulated responses were abnormal in the absence of CPEB3. Using the chemical LTD (c-LTD) protocol, we found that CPEB3 KO neurons displayed morphological and biochemical changes that were slower than those of WT neurons following brief exposures to NMDA. Moreover, two trains of HFS-induced LTP could not be erased and depotentiated in the SC-CA1 neurons of the KO hippocampus when 3 min of weak 5-Hz stimulation was applied to induce depotentiation (DPT). Unlike LTD, which reduces the efficacy of naive synapses, DPT is a process that suppress the strength of previously potentiated synapses (Wagner and Alger, 1996). Both results indicate that certain types of synaptic depression are defective without CPEB3. Using the c-LTD-treated WT and KO neurons, we found that NMDA-induced calcium/calmodulin-dependent protein kinase II alpha (CaMKIIα) activation (i.e., Thr286 autophosphorylation) was stronger in the KO neurons and that this result was accompanied by augmented Ser831 phosphorylation of GluA1, which is a modification known to increase synaptic AMPAR levels (Roche et al., 1996; Barria et al., 1997; Mammen et al., 1997; Lee et al., 2000). Because the protocol used to trigger DPT also signals through NMDARs, and a brief inhibition of CaMKIIα activity during weak tetanic stimulation successfully induced DPT in the KO hippocampus, the imbalance of NMDAR-mediated calcium signaling and CaMKIIα activation in the KO neurons at least partially accounts for the specific abnormalities observed in synaptic depression.

## MATERIALS AND METHODS

### ANTIBODIES AND CHEMICALS

Antibodies used in the study were: Calcineurin A from GeneTex; GFP from AnaSpec; Calmodulin, CaMKIIα, p-CaMKIIα/Thr286, NR1, NR2A, NR2B, GluA1, p-GluA1/Ser831, p-GluA1/Ser845, PSD95, and synaptophysin (SVP38) from Millipore; β-actin, and α-tubulin form Sigma-Aldrich. The CPEB3 monoclonal antibody has been described before (Chao et al., 2012; Wang and Huang, 2012). Alexa Fluor-conjugated secondary antibodies were obtained from Invitrogen. With the exception of CK59 (Calbiochem), all of the other chemicals were purchased from Sigma-Aldrich.

### ANIMALS AND GENOTYPING

All of the experimental protocols were performed in accordance with the guidelines of the Institutional Animal Care and Utilization Committee and compliant with Taiwan Ministry of Science and Technology guidelines for ethical treatment of animals. C57BL/6 mice were housed under a 12-h light/dark cycle in a climate-controlled room with *ad libitum* access to food and water. All efforts were made to minimize the number of animals used and their suffering. The WT and KO embryos and mice were obtained from heterozygous intercrosses. The genotypes were determined by PCR using tail biopsies and the KAPA mouse genotyping kit (KAPA Biosystems) as described before (Chao et al., 2013).

### PLASMID CONSTRUCTION AND LENTIVIRUS PRODUCTION

The myc-CPEB3 (encoding 1–684 amino acids of human CPEB3) and myc-CPEB3N (encoding 1–427 amino acids of human CPEB3) DNA fragments were excised from their corresponding pcDNA3.1 plasmids (Chao et al., 2012) using NheI and PmeI. The pLL3.7-Syn lentiviral plasmid (gift of M Shen) was digested with EcoRI to remove GFP DNA insert and then dephosphorylated. To create multiple cloning sites in this vector, the two oligonucleotides, 5′-AATTAAGCTGCTAGCGGATCCCCCGGGACCGGTG3′ and 5′-AATTCACCGGTCCCGGGGGATCCGCTAGCAGCTT-3′, were annealed and cloned into EcoRI-linearied pLL3.7-Syn. The resulting plasmid was digested with NheI and SmaI for cloning of the myc-CPEB3 and myc-CPEB3N DNA fragments. Further sequence details of these plasmids are available upon request. HEK293T cells were cultured in Dulbecco’s modified Eagle medium supplemented with 10% fetal bovine serum. Lentivirus particles were generated using Virapower packaging system (Invitrogen) and HEK293T cells following the manufacture’s protocol.

### PRIMARY NEURONAL CULTURES, DNA TRANSFECTION AND LENTIVIRAL INFECTION

Because female KO mice exhibited severely reduced fertility, the embryos of different genotypes were collected from heterozygous matings. To culture CPEB3 WT and KO neurons, the cortices and hippocampi of E17.5 embryos from heterozygous matings were isolated and maintained individually in Hank’s balanced salt solution (HBSS) for 2 h on ice. At the same time, the tails were collected for genotyping. Once the genotypes were determined, the WT and KO cerebral cortices were pooled and digested in papain solution (0.6 mg/ml papain and DNase I, 0.5 mM ethylenediaminetetraacetic acid (EDTA), 0.2 mg/ml cysteine and 1.5 mM CaCl_2_ in HBSS). The dissociated neurons were cultured in Neurobasal medium with B27 supplement. Neurons were plated on poly-L-lysine-coated 18-mm coverslips in a 12-well plate with the density of 3 × 10^5^ cells/well or culture dishes with the density of 2 × 10^6^ cells/60-mm dish and 8 × 10^6^ cells/100-mm dish (Chao et al., 2012). Delivery of DNA into the neurons was performed using calcium phosphate transfection or lentiviral infection following previously reported procedures (Chao et al., 2012).

### IMMUNOFLUORESCENCE STAINING, IMAGING ACQUISITION, AND QUANTIFICATION

Neurons at 14 days *in vitro* (DIV) were transfected with the enhanced green fluorescent protein (EGFP) plasmid using calcium phosphate. On DIV 18, the transfected neurons were treated with or without 30 μM NMDA for 3 min and incubated in the culture medium for another 40 min prior to immunostaining using the GFP antibody and Alexa Fluor 488-conjugated secondary antibody. Acquisition of the fluorescent images was performed using a LSM510META confocal microscope (Carl Zeiss) with a Plan-Apochromat 63X/1.25 NA oil objective lens. Each image consisted of a stack of 7–9 Z-series images at a spacing of 0.5 μm. The images were quantified using the MetaMorph software and then exported into Excel and GraphPad Prism for analyses. Approximately 2,000 spines within 20-μm dendritic segments 30 μm away from the somas of 20 neurons were measured in each group (Chao et al., 2013).

### SYNAPTOSOME PREPARATION FROM CULTURED NEURONS

The CPEB3 WT and KO (DIV12–13) neurons in 100-mm dishes were infected with the lentivirus expressing EGFP (as a negative control), myc-CPEB3 or myc-CPEB3N. The infected neurons were stimulated with or without a 3-min pulse of 20 μM NMDA on DIV16–17 and then harvested at the indicated time to obtain the total and synaptosomal lysates for Western blotting. Similar approaches were also employed to harvest total, post-nuclear and synaptosomal lysates from DIV17–18 non-infected WT and KO neurons. Briefly, the neurons were homogenized in 1 ml sucrose buffer [10 mM HEPES pH 7.5, 1.5 mM MgCl_2_, 320 mM sucrose, 5 mM EDTA, 5 mM DTT, 100 μM PMSF, 10 μM MG132, 1X protease and phosphatase inhibitors (Roche)] using a Dounce homogenizer. The homogenates were centrifuged at 700 x*g* for 10 min at 4°C to remove the nuclei and cell debris. The post-nuclear supernatant was then centrifuged again at 9,250 × *g* for 15 min to obtain the pellet containing the crude synaptosome.

### SLICE PREPARATION AND FIELD RECORDING

Either WT or KO male mice (2–3-month-old) obtained from heterozygous mating were anesthetized with isoflurane and decapitated soon after the disappearance of all corneal reflexes. The brain was immediately isolated and placed in ice–cold artificial cerebral spinal fluid (aCSF, 124 mM NaCl, 4.4 mM KCl, 1 mM NaH_2_PO_4_, 1.3 mM MgSO_4_, 10 mM D-glucose, 26 mM NaHCO_3_, 2.5 mM CaCl_2_, and 0.5 mM ascorbic acid, pH 7.4) and oxygenated with 95% O_2_ and 5% CO_2_. Transverse hippocampal slices (400 μm thick) were prepared using a microslicer (DTK-1000, DSK, Japan) and recovered in a submerged holding chamber perfused with oxygenated aCSF at 28°C for at least 2 h. The slices were then transferred to an immersion-type chamber perfused with aCSF at a flow rate of 2–3 ml/min and maintained at 30 ± 1°C to record the field excitatory post-synaptic potentials (fEPSPs). An incision was made between the CA1 and CA3 areas to remove the afferent input from CA3. A concentric bipolar tungsten stimulating electrode (No. 795500, A-M Systems) was placed in the stratum radiatum near the CA2 region and a glass recording microelectrode (No. 615500, A-M Systems) filled with aCSF was placed in the stratum radiatum of the CA1 region. The input–output responses were measured using stimulus intensities from 20 to 110 μA. The baseline stimulation (0.017 Hz, 0.1 ms pulse duration, biphasic) was adjusted to evoke 30–40% of the maximal response for LTP. The quantification of the synaptic transmission strength was measured using the slope of the fEPSP (using the minimum slope over 10–90% of the rising phase). A stable baseline was acquired 20–30 min before stimulation. LTP was evoked by HFS with two trains of 100 Hz (20-s inter-trial interval). Five minutes after LTP induction, DPT was induced by low frequency stimulation with the following parameters: 5 Hz for 3 min, 5 Hz for 8 min, 1 Hz for 15 min, or 2 Hz for 10 min. The specific CaMKIIα inhibitor 10 mM CK59 in dimethyl sulfoxide was diluted to 10 μM in the aCSF just before use. CK59 was applied by switching the perfusion from control aCSF to CK59-containing aCSF for 3 min at 5 min after LTP induction. The average fEPSP slope measured at the indicated time after stimulation was used for statistical comparisons with Student’s *t*-tests.

## RESULTS

### CPEB3 KO NEURONS RESPOND MORE SLOWLY TO C-LTD-INDUCED MORPHOLOGICAL AND BIOCHEMICAL CHANGES

**FIGURE 1:**
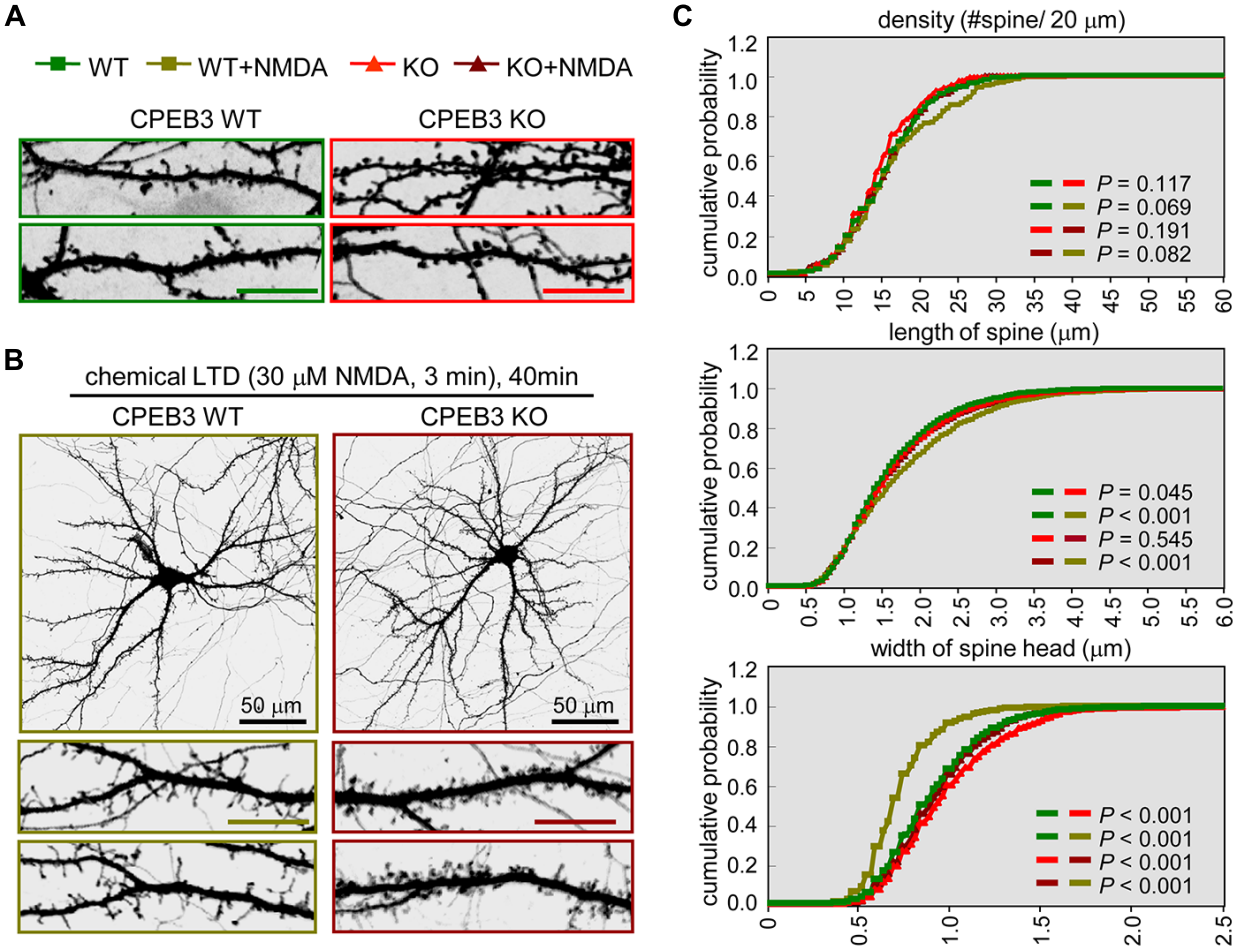
**The CPEB3 KO neurons with enlarged spine heads displayed retarded morphological changes under c-LTD.** The WT and KO neurons were transfected with the EGFP plasmid on DIV14 and treated on DIV18 **(A)** without or **(B)** with 30 μM NMDA for 3 min and then incubated for 40 min prior to fixation for GFP immunostaining. The images and quantified spine data in **(C)** were labeled with different colors, WT: green, WT + NMDA: moss green, KO: red, KO + NMDA: maroon. Representative images of whole neurons and dendritic spine areas are shown. The scales are 10 μm unless otherwise denoted. **(C)** Quantification of the dendritic spine morphologies. Approximately 20 pyramidal neurons in each group were collected from three independent cultures and analyzed using the MetaMorph software. The cumulative probability curves for density, length and width of dendritic spines in each group (∼2,000 spines) were plotted and analyzed for significant differences between groups using Student’s *t*-tests.

**FIGURE 2:**
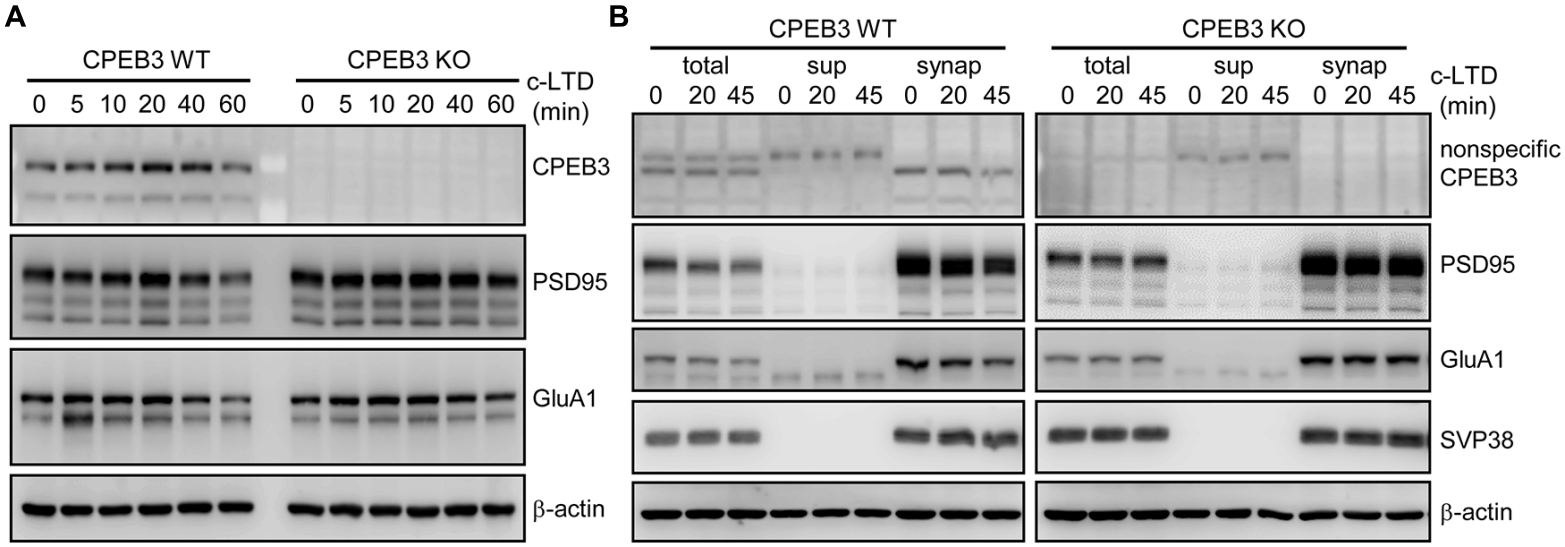
**Slow degradation of PSD95 and GluA1 in c-LTD-treated CPEB3 KO neurons. (A)** The DIV17–18 WT and KO neurons were treated with or without 3 min of 20 μM NMDA and harvested at different time points for Western blotting. **(B)** Similar to **(A)**, in addition to the total lysates, the subcellular fractions (sup, post-nuclear supernatant; synap, synaptosome) were isolated and used for immunoblotting with the denoted antibodies.

In our previous study, we identified that the dendritic spines of CPEB3 KO pyramidal neurons are slightly enlarged. The protein levels of PSD95 and the subunits of AMPAR (i.e., GluA1) and NMDAR (i.e., NR1) begin to increase by ∼15–20% in the KO neurons cultured for 17–20 DIV (Chao et al., 2013). To examine whether such cellular changes in the KO neurons affect activity-regulated responses, we used a protocol known to evoke c-LTD in cultured neurons and young (3- to 4-week-old) hippocampal slices via a brief exposure to low concentrations (20–30 μM) of NMDA that do not cause significant neuronal death (Lee et al., 1998; Beattie et al., 2000; Ashby et al., 2004; Shehata et al., 2012; Wang and Huang, 2012). The cortical/hippocampal neurons were cultured using WT and KO E17.5 embryos obtained from heterozygous matings. DIV14 neurons transfected with a plasmid expressing EGFP were treated with or without a 3-min pulse of NMDA on DIV18 and then fixed 40 min later for GFP immunostaining. Similar to our previous findings (Chao et al., 2013), the CPEB3 deficiency significantly affected the width of the spine heads (representative spine images are shown in **Figure 1A**, and the quantified results are shown in **Figure 1C**, WT: green line, KO: red line). Strikingly, the c-LTD treatment induced a dramatic reduction in spine width in the WT neurons but only induced mild changes in the KO neurons (representative images are shown in **Figure 1B**, and the quantified results are shown in **Figure 1C**, WT + NMDA: moss green line, KO + NMDA: maroon line). This c-LTD protocol has previously been shown to induce PSD95 degradation (Colledge et al., 2003) and AMPAR endocytosis (Lee et al., 1998; Beattie et al., 2000; Ashby et al., 2004) and degradation (Bi et al., 1997; Gellerman et al., 1997; Yuen et al., 2007). A delay in the c-LTD-induced morphological changes in the spines of KO neurons motivated us to examine whether the degradations of PSD95 and AMPAR were affected in the CPEB3-deficient neurons. Thus, the 17–18 DIV WT and KO neurons stimulated with NMDA for 3 min were harvested at different time points for immunoblotting (**Figure 2A**). The PSD95 level of the KO neurons was slightly elevated (time point 0) and declined more slowly following NMDA stimulation. Similarly, when subcellular fractions were used, both the total and the synaptosomal pools of PSD95 and GluA1 were reduced slowly in the NMDA-stimulated KO neurons (**Figure 2B**).

### IMPAIRED DEPOTENTIATION IN THE CPEB3-DELETED HIPPOCAMPAL SLICES

**FIGURE 3:**
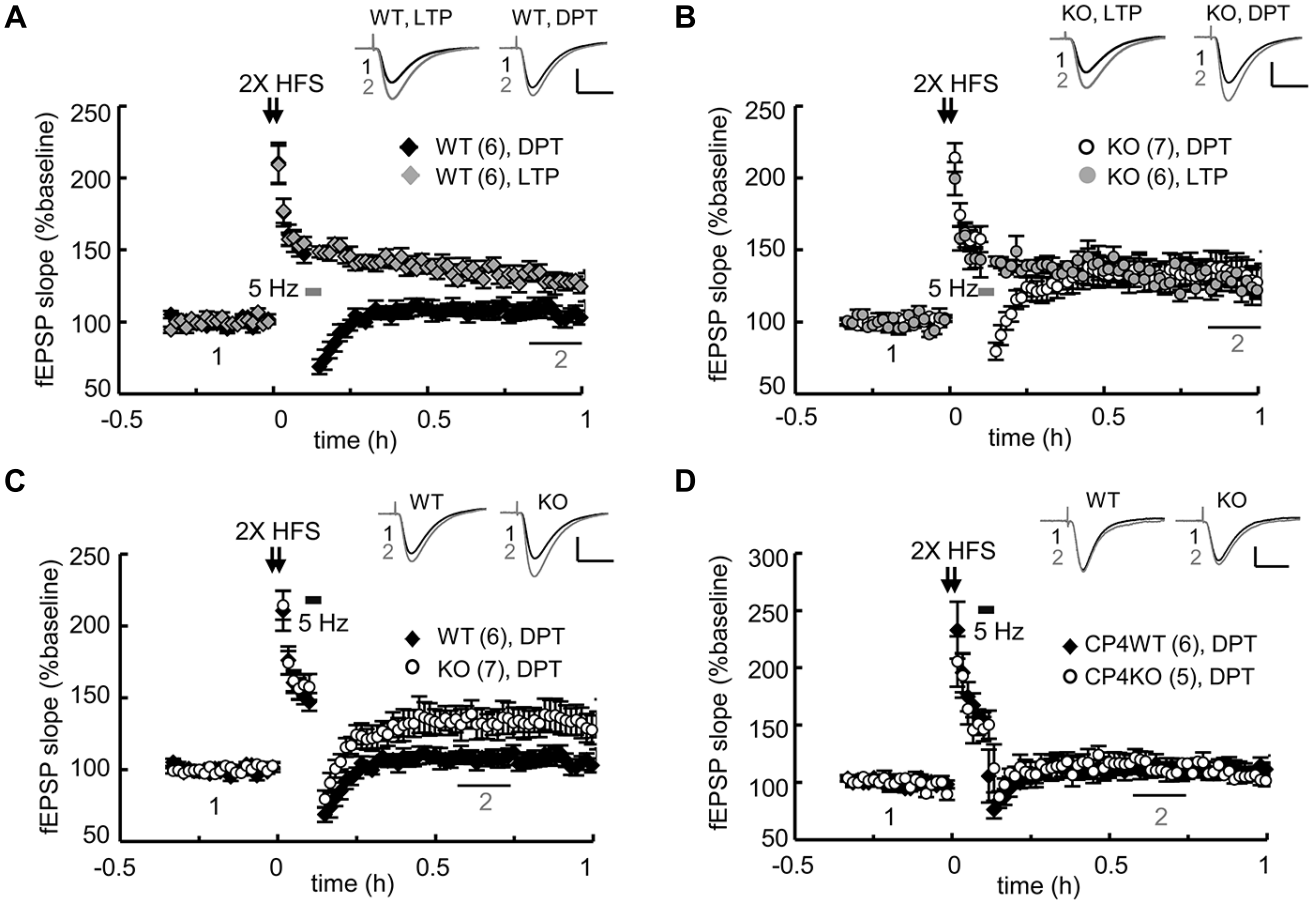
**Genetic ablation of CPEB3 impaired depotentiation in the SC*-*CA1 pathway of hippocampal slices.** The slices prepared from CPEB3 **(A)** WT and **(B)** KO mice were stimulated with two trains (2X) of HFS to induce LTP. To erase the 2X HFS-evoked LTP, 3 min of 5-Hz tetanus was applied 5 min after the HFS to induce DPT. Only DPT but not LTP was defective in the KO slices (WT, LTP: 128.80 ± 5.12%, DPT: 106.46 ± 5.28%, *P* < 0.01 at 50–60 min; KO, LTP: 127.04 ± 8.61%; DPT: 134.12 ± 10.57%, *P* = 0.26 at 50–60 min). **(C)** Depotentiation was impaired in the adult CPEB3KO mice (WT: 107.72 ± 4.71%, KO: 133.36 ± 9.35%, *P* < 0.01 at 30–40 min after stimulation). **(D)** Deletion of cpeb4 gene did not affect DPT (CP4WT: 112.22 ± 6.63%; CP4KO: 115.87 ± 7.45%, *P*= 0.20 at 30–40 min after stimulation). The numbers in parentheses represent the numbers of recorded slices isolated from 5 to 6 male mice. All of the data are expressed as the mean ± SEM. The statistics were performed with Student’s *t*-tests. The traces represent the baseline (black line, 1) and the indicated time after stimulation (gray line, 2). Calibration: 0.5 mV, 20 ms.

The slow morphological and biochemical changes in the NMDA-stimulated KO neurons were not the result of defective NMDAR signaling because the calcium influx through NMDARs is higher in KO neurons due to the elevated expression of the receptors (Chao et al., 2013). These aberrant c-LTD-induced responses suggest that some types of NMDAR-dependent synaptic depression might be affected in the KO hippocampal slices. Our previous study showed that the LTD induced by LFS (1 Hz for 15 min) is normal but that evoked by PP-LFS (50 ms interpulse interval, 1 Hz for 15 min) is enhanced in young (3–4-week-old) KO hippocampal slices (Chao et al., 2013). Although both forms of LTD in young mice require the activation of NMDARs, the slow degradations of PSD95 and GluA1 observed in the c-LTD-treated KO neurons cannot account for the mechanism that underpins the facilitation of PP-LFS-evoked LTD in the KO slices. PP-LFS is a stronger induction protocol than LFS and can evoke NMDAR-independent LTD in adult slices (Oliet et al., 1997; Kemp et al., 2000), but this form of LTD in adult (2–3-month-old) KO slices is normal (Chao et al., 2013). NMDA-induced c-LTD is age-dependent and exhibits robust depression in hippocampal slices prepared from young animals (Lee et al., 1998). Nevertheless, we were more interested in identifying a defective type of NMDAR-dependent synaptic depression in the adult KO slices because the behavioral phenotypes were identified using adult mice (Chao et al., 2013). The other type of synaptic depression, DPT, is also known as “reversal of LTP” and shares many resemblances to LTD, including the reduction of synaptic AMPARs. Recent studies suggest that DPT might be one of the cellular mechanisms that underlies memory extinction (Kim et al., 2007; Hong et al., 2009) in the amygdala and the suppression of previously established spatial memories in the hippocampus (Xu et al., 1998; Zhang et al., 2011; Qi et al., 2013). Because CPEB3 null mice exhibit slower responses during fear extinction in contextual fear conditioning tests and reversal spatial learning in the water maze (Chao et al., 2013), we examined whether DPT was affected in the adult KO hippocampal slices. Two trains of HFS (2X HFS) induced LTP in both WT (**Figure 3A**) and KO slices (**Figure 3B**) to a comparable amplitude, but the reversal of LTP triggered by applying 3 min of 5-Hz weak tetanus (Woo and Nguyen, 2003; Jouvenceau et al., 2006) was evidently impaired in the KO slices (**Figures 3B,C**). In contrast, this type of DPT was normal in the slices prepared from CPEB4 KO mice, which did not exhibit aberrant hippocampus-dependent learning and memory (Tsai et al., 2013; **Figure 3D**). Therefore, ablation of the *cpeb3* but not the *cpeb4* gene impaired synaptic DPT in the SC-CA1 neurons.

### NOT ALL FORMS OF SYNAPTIC DEPOTENTIATION ARE DEFECTIVE IN THE CPEB3 KO HIPPOCAMPUS

**FIGURE 4:**
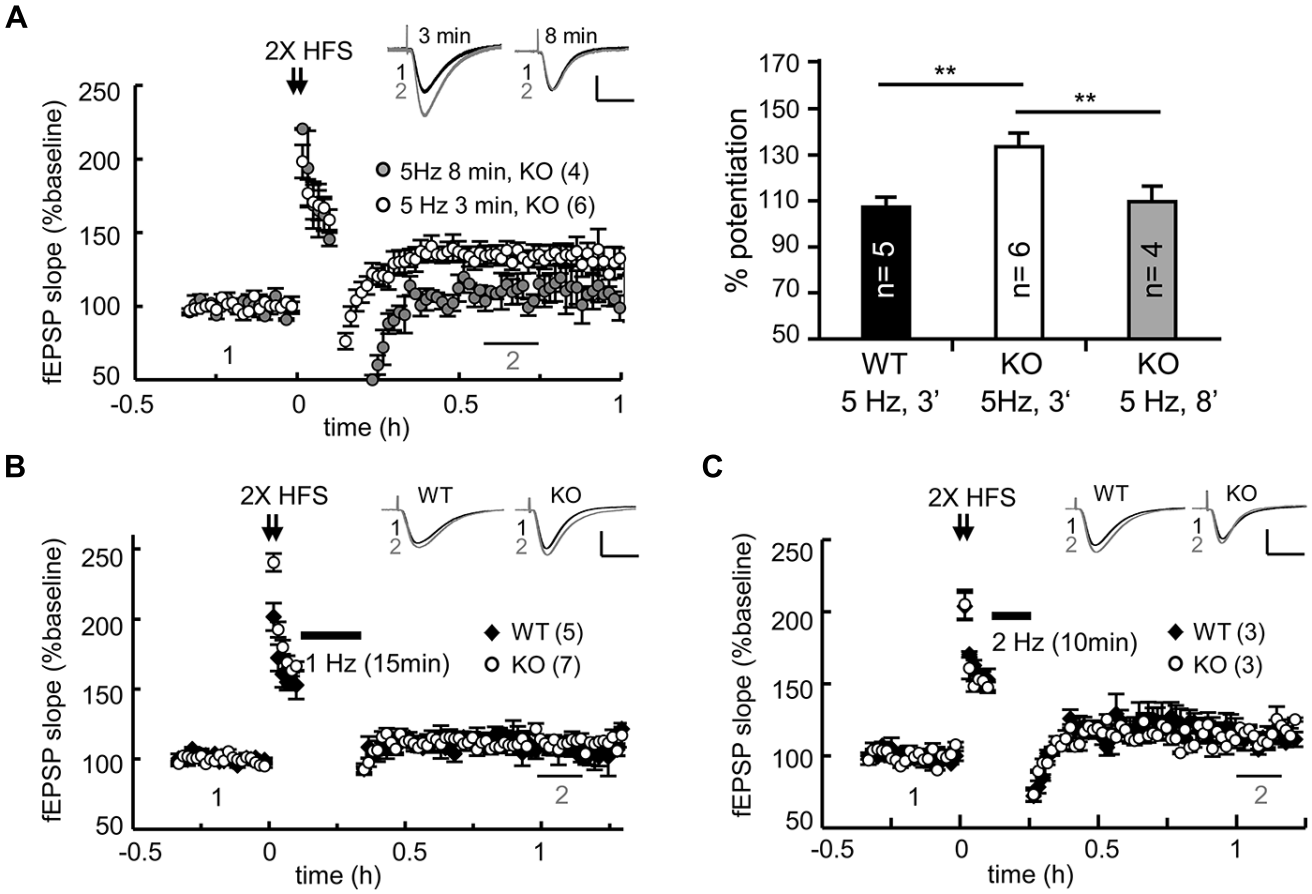
**CPEB3 deficiency did not affect all forms of DPT. (A)** Prolonged 5-Hz stimulation successfully reversed 2X HFS-evoked LTP in the KO slices. The bar graphs show the % of potentiation at 30–40 min after the application of a weak stimulation (3 min of 5 Hz, WT: 108.54 ± 3.31%, KO: 134.18 ± 5.73%; 8 min of 5 Hz, KO: 110.44 ± 6.13%). Five minutes after 2X HFS, the DPT induced by **(B)** 15 min of 1-Hz (WT: 107.32 ± 5.54%, KO: 111.13 ± 5.48%, *P* = 0.11 at 60–70 min) or **(C)** 10 min of 2 Hz stimulus (WT: 113.21 ± 5.39%, KO: 115.61 ± 3.18%, *P*= 0.23 at 60–70 min) exhibited no significant difference between the WT and KO groups. The numbers in parentheses represent the numbers of recorded slices isolated from 5 to 6 male mice. All of the data are expressed as the mean ± the SEM. The statistics were performed with using Student’s *t*-tests. **P < 0.01. The traces represent the baseline (black line, 1) and the indicated time after stimulation (gray line, 2). Calibration: 0.5 mV, 20 ms.

Regardless of whether it is LTD or DPT, synaptic depression depends on the reduction of synaptic AMPARs via various signaling-dependent molecular pathways that control their endocytosis and degradation. Could the elevated expression of PSD95 and AMPARs in the KO neurons result in slower degradation of AMPARs and consequently defective DPT? Could conditioning (i.e., 2X HFS in this case)-induced synaptic potentiation be erased in the KO slices if the duration or strength of a weak stimulus is changed? We examined the DPT evoked by various low frequency stimulations, including 8 min of 5-Hz tetanus (for a total of 2400 pulses), 15 min of 1-Hz tetanus (for a total of 900 pulses) and 10 min of 2-Hz tetani (for a total of 1200 pulses). As a control, we always included one recording of 3 min of 5-Hz-evoked DPT in every slice preparation. Prolonged application of 5-Hz tetanic stimulation was able to reverse potentiation in the KO slices to a level comparable to that observed in the WT slices (**Figure 4A**, WT: 3 min, 108.54 ± 3.31%; KO: 3 min, 134.18 ± 5.73% and 8 min, 111.13 ± 8.85% at 30–40 min after stimulation). Similarly, the DPT induced by 15 min of 1-Hz (**Figure 4B**) or 10 min of 2-Hz tetanic stimulation (**Figure 4C**) was normal in the CPEB3-deficient slices. Thus, only a selective type of DPT in the SC-CA1 neurons was affected by the absence of CPEB3.

### ELEVATED CAMKIIα ACTIVATION AND CAMKIIα-PHOSPHORYLATED SERINE 831 OF GluA1 IN THE C-LTD-TREATED KO NEURONS

**FIGURE 5:**
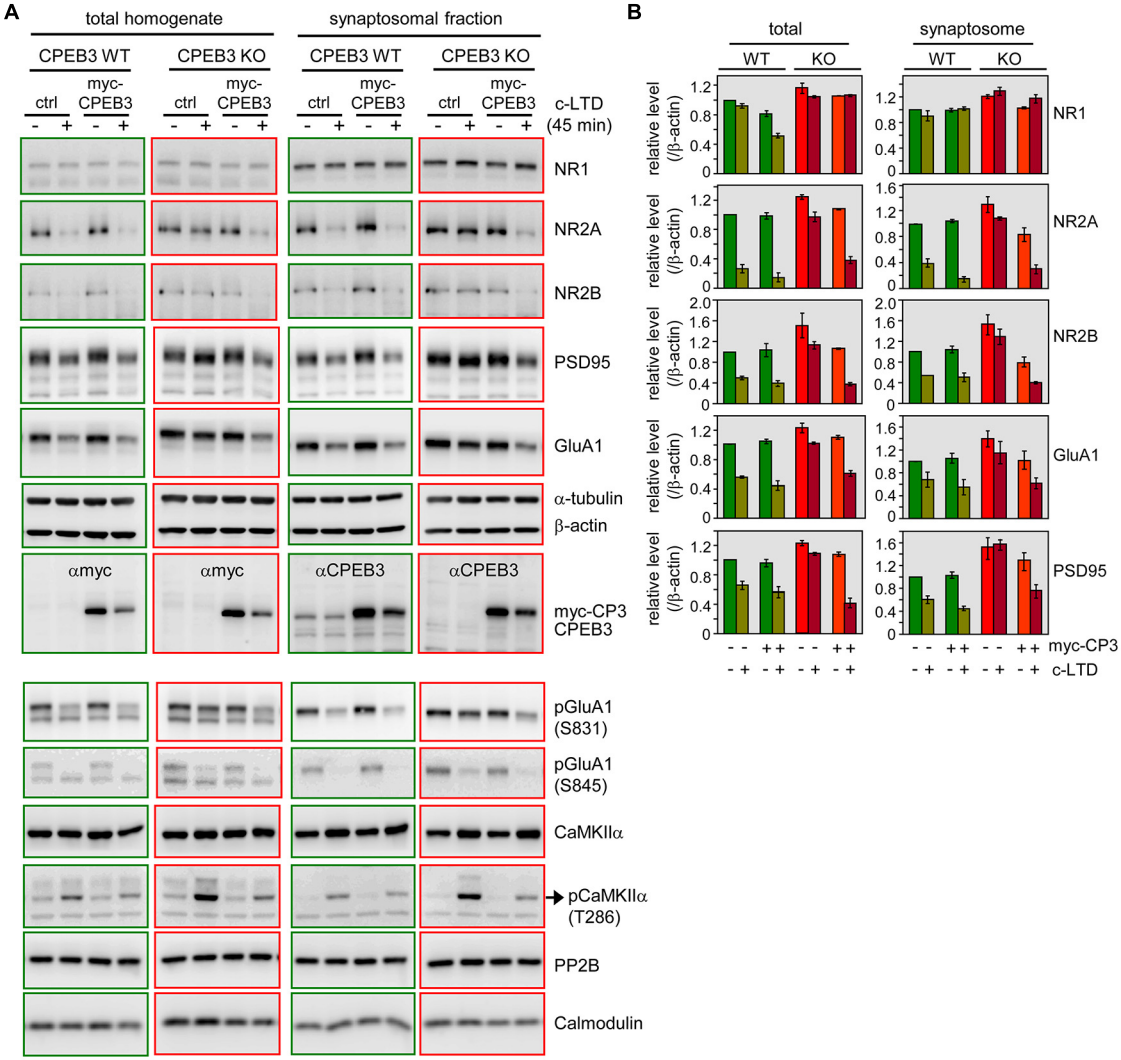
**Delayed c-LTD-induced biochemical changes in the KO neurons were rescued by ectopically expressing CPEB3. (A)** C-LTD induced decreases in the levels of NR2A, NR2B, GluA1, and PSD95 proteins, which declined more slowly in the KO neurons and could be rescued by ectopically expressing myc-CPEB3. The WT and KO (DIV12–13) neurons were infected with lentivirus expressing EGFP (ctrl) or myc-CPEB3. The infected neurons at DIV16–17 were stimulated with or without a 3-min pulse of 20 μM NMDA and then harvested 45 min later to obtain total and synaptosomal lysates for Western blotting. Alpha-tubulin and β-actin served as the loading controls. **(B)** The quantified immunoblot signals of the indicated proteins are expressed as the relative ratios, and the signal in the untreated WT neurons was arbitrarily set to one. The results from two independent preparations of cultured neurons were analyzed and are expressed as the mean ± SEM. Other molecules, including phosphorylated GluA1 (pGluA1) at Ser831 and Ser845, CaMKIIα, phosphorylated CaMKIIα (pCaMKIIα) at Thr286, PP2B (calcineurin) and calmodulin, were further examined using one of more abundant prepared samples.

The stimulation protocols used to evoke the aforementioned DPT in the SC-CA1 pathway require signaling through NMDARs and phosphatase activity (Zhuo et al., 1999; Huang et al., 2001; Jouvenceau et al., 2003, 2006). Pharmacological interventions and genetic experiments have indicated that 10 min of 2-Hz and 15 min of 1-Hz-induced DPT depend more on the activities of protein phosphatase 1 (PP1) and PP2A, whereas 3 min of 5-Hz-triggered DPT relies on PP2B (Zhuo et al., 1999; Huang et al., 2001; Jouvenceau et al., 2003). Thus, the signaling cascades that are activated to reverse potentiated synaptic responses differ between various forms of DPT. To further delineate the molecular defects underlying the specific type of DPT, we collected synaptic areas between stimulating and recording electrodes from depotentiated WT and KO slices at 0, 15, 30, and 60 min for immunoblotting of GluA1 and PSD95. Because it is impossible to know whether synapses in the isolated tissues are all equally depotentiated, we observed variable results from three independent experiments to draw our conclusions. Moreover, the minute amounts of isolated hippocampal tissues also limited the immunodetection of changes in the expression or phosphorylation levels of various synaptic proteins. In contrast, the 3-min pulse of NMDA induced c-LTD at all synapses throughout all neurons instead of being confined to a small fraction of synapses near the stimulating electrode. The common endpoint of c-LTD and DPT is the reduction of synaptic AMPARs. Additionally, c-LTD is sensitive to PP2B but not PP1/PP2A inhibitors (Kameyama et al., 1998). Thus, the defective type of DPT identified from the KO slices seems to share a molecular resemblance with the retarded c-LTD responses of the cultured KO neurons. We then used c-LTD-treated WT and KO neurons to further explore other aberrant molecular changes. Furthermore, to determine whether the exogenous expression of myc-CPEB3 could suppress the elevated protein levels of PSD95, GluA1 and NMDAR subunits (NR1, NR2A, and NR2B) and consequently rescue the c-LTD-induced biochemical defects in the KO neurons, WT and KO neurons at DIV12–13 were infected with the lentivirus expressing EGFP (ctrl) or myc-CPEB3 (myc-CP3) and subsequently harvested at DIV16–17 to obtain total and synaptosomal lysates for immunoblotting (**Figure 5A**). The total and synaptic levels of NR1, NR2A, NR2B, GluA1, and PSD95 were slightly elevated in the CPEB3 KO neurons, and similar observation have been made with 3-month-old CPEB3 KO brains (Chao et al., 2013). Notably, the most salient biochemical difference between the WT and KO neurons was their response to c-LTD-induced GluA1 and PSD95 degradation. Not only were GluA1 and PSD95 decreased, but NR2A and NR2B were also significantly reduced in the WT neurons and the WT and KO neurons that ectopically expressed myc-CPEB3 within 45 min after the 3-min pulse of NMDA. In contrast, these decreases were less obvious in the KO neurons (**Figures 5A,B**). The slow c-LTD-induced changes in the KO neurons could also be attributed to aberrant NMDAR signaling. As shown in our previous study, the calcium influx mediated through the opening of NMDARs in the KO neurons is greater than that in WT neurons. This defect could be rescued by ectopically expressing myc-CPEB3 presumably via the downregulation of the expression of NMDARs (Chao et al., 2013). Thus, we next examined the NMDAR downstream signaling molecules CaMKIIα and PP2B (i.e., calcineurin). Although the expression levels of these molecules were comparable between the WT and KO neurons with and without c-LTD treatment, the Thr286 autophosphorylation signal of CaMKIIα (i.e., the indicator of its kinase activity) was evidently increased in the NMDA-treated KO neurons. In accordance with this finding, the level of GluA1 (p-GluA1) phosphorylated at Ser831 by CaMKIIα was also elevated in the KO neurons. In contrast, the amount of p-GluA1 at Ser845 by protein kinase A was slightly upregulated in the KO neurons.

### TRANSIENT SUPPRESSION OF CAMKIIα ACTIVITY RESCUES THE DPT DEFECT IN THE CPEB3-DEFICIENT HIPPOCAMPUS

**FIGURE 6:**
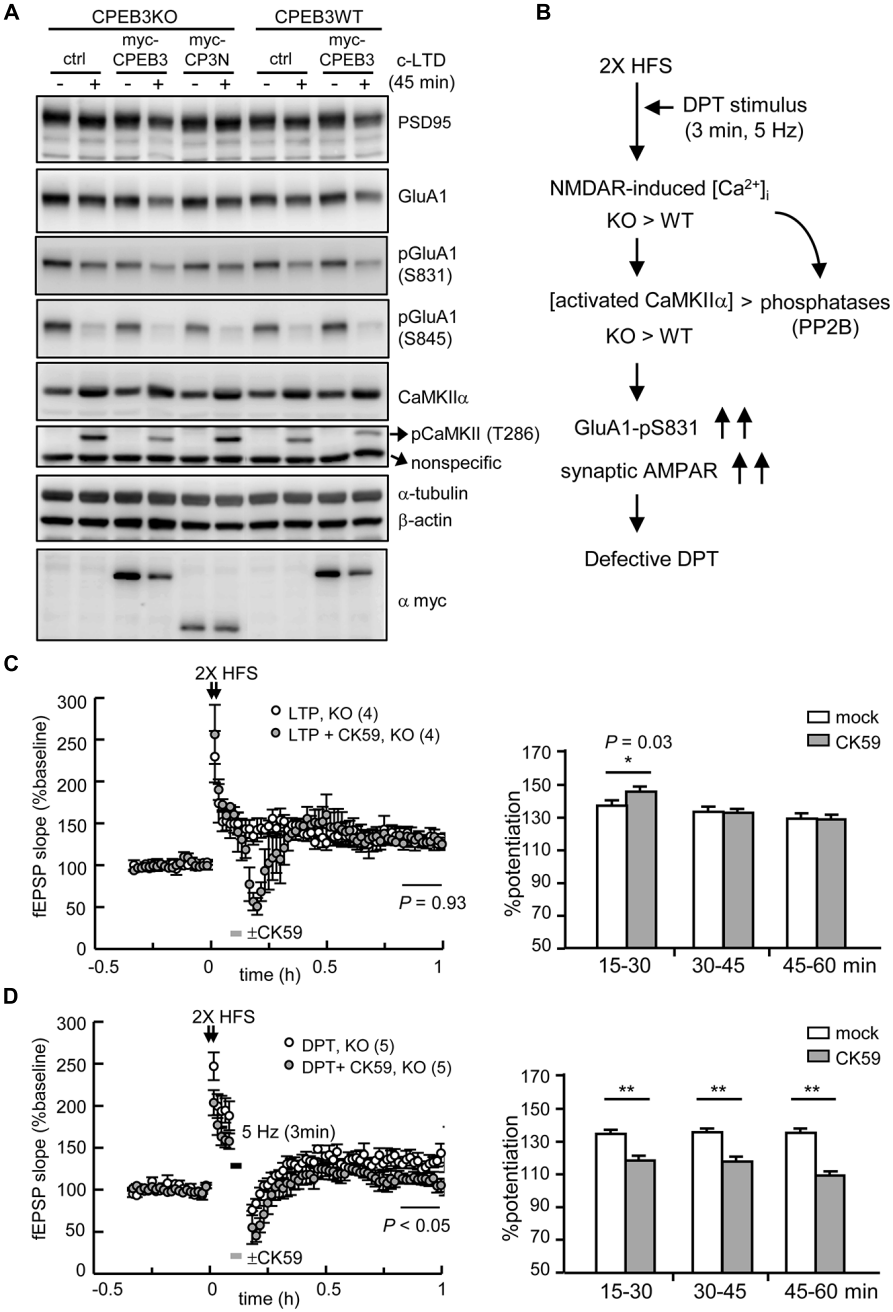
**The defective form of DPT in the CPEB3-deficient hippocampus was rescued by the transient inhibition of CaMKIIα activity. (A)** The neurons infected with or without the lentivirus expressing myc-CPEB3 or the N-terminus of CPEB3 (myc-CP3N) were stimulated with or without a 3-min pulse of 20 μM NMDA and then harvested 45 min later for Western blotting. **(B)** The results from c-LTD-treated KO neurons suggested that the DPT defect in the KO hippocampal slices might have been caused by elevated NMDAR-mediated calcium influx, which shifted the balances between calcium/calmodulin-dependent kinase and phosphatase, i.e., CaMKIIα and calcineurin (PP2B), respectively. Consequently, the increased Ser831 phosphorylation of GluA1 failed to decrease the synaptic AMPAR level to induce DPT. **(C)** The 2X HFS-induced LTP in the KO slices was not affected by the 3-min application of the CaMKIIα inhibitor CK59 (10 μM; mock: 130.14 ± 7.81%, CK59: 130.41 ± 6.41%, *P* = 0.93 at 50–60 min). **(D)** The application of CK59 during the 3-min of 5-Hz stimulation facilitated DPT in the KO slices (mock: 133.44 ± 8.47%, CK59: 111.22 ± 6.47%, *P* < 0.01 at 50–60 min after stimulation). The histograms display the % potentiation during three time frames (15–30, 30–45, and 45–60 min). The numbers in parentheses represent the numbers of recorded slices isolated from 3 to 5 male mice. All of the data are expressed as the mean ± SEM. The statistics in **(C)** and **(D)** were performed with Student’s *t-*tests. Single and double asterisks represent *P* < 0.05 and *P* < 0.01, respectively.

We repeated the rescue experiment with lentiviral delivery of EGFP (ctrl), full length myc-CPEB3 and the N-terminus without the RBD of CPEB3 (myc-CP3N) to cultured neurons. Only ectopically expressed myc-CPEB3 and not myc-CP3N rescued the c-LTD defect in the KO neurons (**Figure 6A**), which suggests that the translational up-expression of such proteins as NMDAR and PSD95 is caused by the loss of the repressor CPEB3, which directly accounted for the slow c-LTD responses. Thus, we wondered whether the impaired DPT induced by 3 min of 5-Hz stimulation also resulted from NMDAR-hyperactivated CaMKIIα because elevated protein but not RNA levels of NMDARs have been previously identified in the adult KO brain (Chao et al., 2013). The imbalance between the activations of kinases and phosphatases, such as CaMKIIα and PP2B, respectively, might consequently increase the phosphorylation of GluA1 at Ser831 and the synaptic retention of AMPARs (**Figure 6B**). If this hypothesis is correct, downregulating CaMKIIα activity with the specific inhibitor CK59 during the 3 min of 5-Hz stimulation should enable DPT in the KO hippocampus. Because CaMKIIα activity is required for LTP induction and maintenance (Silva et al., 1992), the control experiment with a brief incubation with CK59 during 2X HFS-evoked LTP was included. The results revealed that the transient application of CK59 had no effect on LTP maintenance (**Figure 6C**) but rescued DPT impairment (**Figure 6D**), which supports the notion that the hyperactivated CaMKIIα during LFS is responsible for the DPT failure in the KO hippocampus.

## DISCUSSION

This study demonstrates that elevated expression of NMDARs in the absence of the translational repressor CPEB3 contributed to the abnormal c-LTD responses and synaptic DPT partially due to the increased calcium influx through NMDARs to hyperactivate CaMKIIα. Although several forms of LTP and LTD in the adult SC-CA1 KO synapses appear normal (Chao et al., 2013), we identified a specific type of synaptic depression, 3 min of 5-Hz stimulus-evoked DPT, that is impaired without CPEB3. This defect was ameliorated by transiently inhibiting the activity of CaMKIIα. Along with the elevated Thr286 autophosphorylation of CaMKIIα and its substrate GluA1 at Ser831 in the KO neurons under the c-LTD treatment, a slight alteration in the expression level of NMDARs selectively affects some but not all NMDAR-dependent synaptic responses.

Memory involves modulations of synaptic plasticity including the abilities of synapses to strengthen (i.e., potentiation) or weaken (i.e., depression) over time in response to connectivity changes. Although electrophysiology is a common approach for assessing possible synaptic defects in mice with aberrant memories, a wealth of studies of transgenic and KO mice has demonstrated that the causal relations between LTP and memory enhancement and LTD and memory impairment are not always established. For example, the mice expressing a mutant PSD95 without functional synaptic PSD95 exhibit potentiated LTP and the absence of LTD, but their learning and memory abilities are impaired (Migaud et al., 1998). Although many factors, such as the age of mice and stimulus conditions used in behavioral and electrophysiological studies can contribute to these discrepancies, hippocampal slice recording remains a powerful tool to identify abnormalities in synaptic plasticity with high spatiotemporal resolution that cannot be achieved by cellular and biochemical approaches. Taking our study for example, 900 pulses of stimuli given in a pattern of 3 min at 5 Hz instead of 15 min at 1 Hz identified DPT defects in the absence of CPEB3.

Long-term depression and DPT occur commonly via the removal of AMPARs from the postsynaptic membrane, but the underlying mechanisms that increase the endocytosis and degradation of AMPARs in response to specific stimulation are very diverse and extremely complex. The regulatory mechanisms vary between distinct subunits of AMPARs, including GluA1 to GluA4. In this study, we focused on the well-characterized GluA1 subunit in the hippocampus. In addition to phosphorylation modification, S-palmitoylation and S-nitrosylation have been recently identified in GluA1 and play roles in the regulation of the dynamic trafficking of AMPARs in and out of synapses (Lu and Roche, 2012; Selvakumar et al., 2013). Moreover, the proteasome, lysosome and other proteases, such as calpain, have been reported to reduce synaptic AMPARs via the degradation of receptors or other auxiliary proteins that facilitate the synaptic targeting of AMPARs (see reviews in Anggono and Huganir, 2012; Huganir and Nicoll, 2013). Phosphorylation and dephosphorylation of the AMPAR GluA1 subunit, especially at Ser831 and Ser845, are critical for hippocampal LTP and LTD and for the synaptic plasticity that underlies learning and memory (Lee et al., 2003; Crombag et al., 2008). DPT and LTD share some biochemical properties, such as a dependence on phosphatase activity to trigger the dephosphorylation and degradation of synaptic AMPARs (Huang et al., 2001; Kim et al., 2007). Nevertheless, it has been reported that LTD induction in naive synapses causes Ser845 dephosphorylation, while DPT leads to the Ser831 dephosphorylation (Lee et al., 2000). Moreover, NMDAR-dependent DPT but not LTD can be easily induced in the adult hippocampus and requires priming stimulation to first potentiate synapses. Interestingly, the DPT defect in the CPEB3-deficient hippocampus was only detected following a specific pattern of stimulation, which suggests that CaMKIIα-hyperphosphorylated GluA1 at Ser831 could be reversed depending on LFS-induced differential phosphatase signaling. For example, 10 min of 2-Hz and 15 min of 1-Hz-induced DPT depends more on the activities of PP1 and PP2A, whereas 3 min of 5 Hz-triggered DPT relies on PP2B (Zhuo et al., 1999; Huang et al., 2001; Jouvenceau et al., 2003). The Thr286 autophosphorylation status of CaMKIIα is critical for learning and memory and is also regulated by PPs. Specifically, PP1 is a major phosphatase that is responsible for the dephosphorylation of CaMKIIα (Genoux et al., 2002). Thus, the imbalance of kinase and phosphatase signaling in the CPEB3-deficient hippocampus only prevails depending on how the NMDARs are activated. In addition to LFS-activated phosphatase signaling, the priming synaptic activity also affects the reversibility of the response because 4X HFS-induced protein synthesis-dependent LTP cannot be depotentiated by 3 or 6-min of 5-Hz stimulation (Woo and Nguyen, 2003). The identities of these newly synthesized proteins that confer synaptic immunity to DPT are currently unknown. Perhaps molecules such as AMPARs, NMDARs and CaMKIIα can be further investigated with this stimulation paradigm since all of them can be synthesized via translational regulation. Similar to CPEB3 KO mice, the genetic deletion of eIF-4E binding protein 2 (4E-BP2) in mice upregulates translation of GluA1 and GluA2 RNAs (Ran et al., 2013). Expressions of CaMKIIα and NR2A could be regulated through CPEB1-mediated polyadenylation-activated translation, respectively, in visual cortex of dark reared rats following exposure to light (Wu et al., 1998) and under the protocol used to stimulate chemical LTP in neurons (Udagawa et al., 2012; Swanger et al., 2013). Translation of CaMKIIα mRNA is enhanced in the KO mice without the expression of fragile X mental retardation 1 (Zalfa et al., 2003) or PABP-interacting protein 2A (Khoutorsky et al., 2013). Furthermore, miR-124 (Dutta et al., 2013), miR-132, miR-181a (Saba et al., 2012), and miR-223 (Harraz et al., 2012) were reported to directly or indirectly influence the protein levels of some subunits of AMPARs and/or NMDARs.

Bi-directional control of synaptic strength is important for dynamic behavioral changes during different phases of memory. Activity-dependent synaptic modifications are required not only for memory consolidation but also for memory extinction. Moreover, novelty acquisition of spatial information can induce the reversal of previously established LTP in the hippocampus (Xu et al., 1998). Memory extinction and spatial novelty acquisition are processes that suppress or adjust the previously established memory for behavioral adaptation to the changing environments. At the cellular level, the reversal of synaptic potentiation (i.e., DPT) is believed to be important for neurons to acquire new information and to prevent synaptic saturation (Xu et al., 1998; Kim et al., 2007; Hong et al., 2009; Qi et al., 2013). Thus, LFS-induced DPT requires the dephosphorylation and reduction of synaptic AMPARs to weaken synaptic efficacy (Huang et al., 2001; Kim et al., 2007). In our previous study, CPEB3 KO mice exhibited better consolidation of spatial memory in the Morris water maze, but their ability to rapidly acquire new spatial information during the first trial of reversal learning when the platform was relocated to a new position was obviously impaired. However, the KO mice were able to catch up with their WT littermates and behavior normally in the second and third trials of reversal spatial learning. In contextual fear conditioning, the null mice exhibited faster acquisition and slower extinction of short-term fear memory. Given sufficient training, the consolidated long-term fear memory and extinction memory are normal in the KO mice. Thus, the differential memory performances between the WT and KO littermates can be readjusted with additional training. In this study, we further identified that the CPEB3-deleted neurons have increased spine rigidity and reduced synaptic flexibility as evidenced by slow morphological and biochemical responses to the c-LTD condition and defective DPT in the SC-CA1 synapses. Nevertheless, the DPT defects could be ameliorated by prolonging the 5-Hz stimulations from 3 to 8 min or using different LFS protocols. These cellular defects might further explain, at least in part, the behavioral abnormalities, such as the retarded fear extinction and reversal spatial learning observed in the KO mice. In the future, it will be of interest to test whether the CPEB3 KO mice tend to develop post-traumatic stress disorder-like symptoms by increasing the electrical foot shock trials during fear acquisition or reducing the training during fear extinction in contextual and cued fear conditioning behaviors.

## AUTHOR CONTRIBUTIONS

Wen-Hsuan Huang along with Li-Yun Tsai and Ming-Hung Chung performed the electrophysiological experiments. Hsu-Wen Chao conducted all of the molecular and cellular characterizations with the help from Wen-Hsuan Huang to quantify the spine morphology. Yi-Shuian Huang supervised the study and wrote the manuscript with contributions from Wen-Hsuan Huang and Hsu-Wen Chao.

## Conflict of Interest Statement

The authors declare that the research was conducted in the absence of any commercial or financial relationships that could be construed as a potential conflict of interest.

## References

[B1] Anggono V. Huganir R. L. (2012). Regulation of AMPA receptor trafficking and synaptic plasticity. *Curr. Opin. Neurobiol.* 22 461–469. 10.1016/j.conb.2011.12.006 22217700 PMC3392447

[B2] Ashby M. C. De La Rue S. A. Ralph G. S. Uney J. Collingridge G. L. Henley J. M. (2004). Removal of AMPA receptors (AMPARs) from synapses is preceded by transient endocytosis of extrasynaptic AMPARs. *J. Neurosci.* 24 5172–5176. 10.1523/JNEUROSCI.1042-04.2004 15175386 PMC3309030

[B3] Barria A. Derkach V. Soderling T. (1997). Identification of the Ca^2+^/calmodulin-dependent protein kinase II regulatory phosphorylation site in the α-amino-3-hydroxyl-5-methyl-4-isoxazole-propionate-type glutamate receptor. *J. Biol. Chem.* 272 32727–32730. 10.1074/jbc.272.52.32727 9407043

[B4] Beattie E. C. Carroll R. C. Yu X. Morishita W. Yasuda H. Von Zastrow M. (2000). Regulation of AMPA receptor endocytosis by a signaling mechanism shared with LTD. *Nat. Neurosci.* 3 1291–1300. 10.1038/81823 11100150

[B5] Berger-Sweeney J. Zearfoss N. R. Richter J. D. (2006). Reduced extinction of hippocampal-dependent memories in CPEB knockout mice. *Learn. Mem.* 13 4–7. 10.1101/lm.73706 16452649

[B6] Bi X. Chen J. Dang S. Wenthold R. J. Tocco G. Baudry M. (1997). Characterization of calpain-mediated proteolysis of GluR1 subunits of α-amino-3-hydroxy-5-methylisoxazole-4-propionate receptors in rat brain. *J. Neurochem.* 68 1484–1494. 10.1046/j.1471-4159.1997.68041484.x 9084418

[B7] Chao H. W. Lai Y. T. Lu Y. L. Lin C. L. Mai W. Huang Y. S. (2012). NMDAR signaling facilitates the IPO5-mediated nuclear import of CPEB3. *Nucleic Acids Res.* 40 8484–8498. 10.1093/nar/gks598 22730302 PMC3458550

[B8] Chao H. W. Tsai L. Y. Lu Y. L. Lin P. Y. Huang W. H. Chou H. J. (2013). Deletion of CPEB3 enhances hippocampus-dependent memory via increasing expressions of PSD95 and NMDA receptors. *J. Neurosci.* 33 17008–17022. 10.1523/JNEUROSCI.3043-13.2013 24155305 PMC6618447

[B9] Chen P. J. Huang Y. S. (2012). CPEB2-eEF2 interaction impedes HIF-1α RNA translation. *EMBO J.* 31 959–971. 10.1038/emboj.2011.448 22157746 PMC3280548

[B10] Colledge M. Snyder E. M. Crozier R. A. Soderling J. A. Jin Y. Langeberg L. K. (2003). Ubiquitination regulates PSD-95 degradation and AMPA receptor surface expression. *Neuron* 40 595–607. 10.1016/S0896-6273(03)00687-1 14642282 PMC3963808

[B11] Costa-Mattioli M. Sossin W. S. Klann E. Sonenberg N. (2009). Translational control of long-lasting synaptic plasticity and memory. *Neuron* 61 10–26. 10.1016/j.neuron.2008.10.055 19146809 PMC5154738

[B12] Crombag H. S. Sutton J. M. Takamiya K. Lee H. K. Holland P. C. Gallagher M. (2008). A necessary role for GluR1 serine 831 phosphorylation in appetitive incentive learning. *Behav. Brain Res.* 191 178–183. 10.1016/j.bbr.2008.03.026 18455244 PMC2478746

[B13] Darnell J. C. Richter J. D. (2012). Cytoplasmic RNA-binding proteins and the control of complex brain function. *Cold Spring Harb. Perspect. Biol.* 4 a012344. 10.1101/cshperspect.a012344 PMC3405866 22723494

[B14] Dutta R. Chomyk A. M. Chang A. Ribaudo M. V. Deckard S. A. Doud M. K. (2013). Hippocampal demyelination and memory dysfunction are associated with increased levels of the neuronal microRNA miR-124 and reduced AMPA receptors. *Ann. Neurol.* 73 637–645. 10.1002/ana.23860 23595422 PMC3679350

[B15] Gal-Ben-Ari S. Kenney J. W. Ounalla-Saad H. Taha E. David O. Levitan D. (2012). Consolidation and translation regulation. *Learn. Mem.* 19 410–422. 10.1101/lm.026849.112 22904372 PMC3418764

[B16] Gellerman D. M. Bi X. Baudry M. (1997). NMDA receptor-mediated regulation of AMPA receptor properties in organotypic hippocampal slice cultures. *J. Neurochem.* 69 131–136. 10.1046/j.1471-4159.1997.69010131.x 9202303

[B17] Genoux D. Haditsch U. Knobloch M. Michalon A. Storm D. Mansuy I. M. (2002). Protein phosphatase 1 is a molecular constraint on learning and memory. *Nature* 418 970–975. 10.1038/nature00928 12198546

[B18] Harraz M. M. Eacker S. M. Wang X. Dawson T. M. Dawson V. L. (2012). MicroRNA-223 is neuroprotective by targeting glutamate receptors. *Proc. Natl. Acad. Sci. U.S.A.* 109 18962–18967. 10.1073/pnas.1121288109 23112146 PMC3503176

[B19] Hong I. Song B. Lee S. Kim J. Choi S. (2009). Extinction of cued fear memory involves a distinct form of depotentiation at cortical input synapses onto the lateral amygdala. *Eur. J. Neurosci.* 30 2089–2099. 10.1111/j.1460-9568.2009.07004.x 20128847

[B20] Huang C. C. Liang Y. C. Hsu K. S. (2001). Characterization of the mechanism underlying the reversal of long term potentiation by low frequency stimulation at hippocampal CA1 synapses. *J. Biol. Chem.* 276 48108–48117 11679581 10.1074/jbc.M106388200

[B21] Huang Y. S. Kan M. C. Lin C. L. Richter J. D. (2006). CPEB3 and CPEB4 in neurons: analysis of RNA-binding specificity and translational control of AMPA receptor GluR2 mRNA. *EMBO J.* 25 4865–4876. 10.1038/sj.emboj.7601322 17024188 PMC1618119

[B22] Huganir R. L. Nicoll R. A. (2013). AMPARs and synaptic plasticity: the last 25 years. *Neuron* 80 704–717. 10.1016/j.neuron.2013.10.025 24183021 PMC4195488

[B23] Ivshina M. Lasko P. Richter J. D. (2014). Cytoplasmic polyadenylation element binding proteins in development, health, and disease. *Annu. Rev. Cell Dev. Biol.* 30 393–415. 10.1146/annurev-cellbio-101011-155831 25068488

[B24] Jouvenceau A. Billard J. M. Haditsch U. Mansuy I. M. Dutar P. (2003). Different phosphatase-dependent mechanisms mediate long-term depression and depotentiation of long-term potentiation in mouse hippocampal CA1 area. *Eur. J. Neurosci.* 18 1279–1285. 10.1046/j.1460-9568.2003.02831.x 12956726

[B25] Jouvenceau A. Hedou G. Potier B. Kollen M. Dutar P. Mansuy I. M. (2006). Partial inhibition of PP1 alters bidirectional synaptic plasticity in the hippocampus. *Eur. J. Neurosci.* 24 564–572. 10.1111/j.1460-9568.2006.04938.x 16903858

[B26] Kameyama K. Lee H. K. Bear M. F. Huganir R. L. (1998). Involvement of a postsynaptic protein kinase A substrate in the expression of homosynaptic long-term depression. *Neuron* 21 1163–1175. 10.1016/S0896-6273(00)80633-9 9856471

[B27] Kemp N. Mcqueen J. Faulkes S. Bashir Z. I. (2000). Different forms of LTD in the CA1 region of the hippocampus: role of age and stimulus protocol. *Eur. J. Neurosci.* 12 360–366. 10.1046/j.1460-9568.2000.00903.x 10651891

[B28] Khoutorsky A. Yanagiya A. Gkogkas C. G. Fabian M. R. Prager-Khoutorsky M. Cao R. (2013). Control of synaptic plasticity and memory via suppression of poly(A)-binding protein. *Neuron* 78 298–311. 10.1016/j.neuron.2013.02.025 23622065

[B29] Kim J. Lee S. Park K. Hong I. Song B. Son G. (2007). Amygdala depotentiation and fear extinction. *Proc. Natl. Acad. Sci. U.S.A.* 104 20955–20960. 10.1073/pnas.0710548105 18165656 PMC2409248

[B30] Lee H. K. Barbarosie M. Kameyama K. Bear M. F. Huganir R. L. (2000). Regulation of distinct AMPA receptor phosphorylation sites during bidirectional synaptic plasticity. *Nature* 405 955–959. 10.1038/35016089 10879537

[B31] Lee H. K. Kameyama K. Huganir R. L. Bear M. F. (1998). NMDA induces long-term synaptic depression and dephosphorylation of the GluR1 subunit of AMPA receptors in hippocampus. *Neuron* 21 1151–1162. 10.1016/S0896-6273(00)80632-7 9856470

[B32] Lee H. K. Takamiya K. Han J. S. Man H. Kim C. H. Rumbaugh G. (2003). Phosphorylation of the AMPA receptor GluR1 subunit is required for synaptic plasticity and retention of spatial memory. *Cell* 112 631–643. 10.1016/S0092-8674(03)00122-3 12628184

[B33] Lu W. Roche K. W. (2012). Posttranslational regulation of AMPA receptor trafficking and function. *Curr. Opin. Neurobiol.* 22 470–479. 10.1016/j.conb.2011.09.008 22000952 PMC3279598

[B34] Mammen A. L. Kameyama K. Roche K. W. Huganir R. L. (1997). Phosphorylation of the α-amino-3-hydroxy-5-methylisoxazole4-propionic acid receptor GluR1 subunit by calcium/calmodulin-dependent kinase II. *J. Biol. Chem.* 272 32528–32533. 10.1074/jbc.272.51.32528 9405465

[B35] Mendez R. Hake L. E. Andresson T. Littlepage L. E. Ruderman J. V. Richter J. D. (2000). Phosphorylation of CPE binding factor by Eg2 regulates translation of c-*mos* mRNA. *Nature* 404 302–307. 10.1038/35005126 10749216

[B36] Migaud M. Charlesworth P. Dempster M. Webster L. C. Watabe A. M. Makhinson M. (1998). Enhanced long-term potentiation and impaired learning in mice with mutant postsynaptic density-95 protein. *Nature* 396 433–439. 10.1038/24790 9853749

[B37] Oliet S. H. Malenka R. C. Nicoll R. A. (1997). Two distinct forms of long-term depression coexist in CA1 hippocampal pyramidal cells. *Neuron* 18 969–982. 10.1016/S0896-6273(00)80336-0 9208864

[B38] Pavlopoulos E. Trifilieff P. Chevaleyre V. Fioriti L. Zairis S. Pagano A. (2011). Neuralized1 activates CPEB3: a function for nonproteolytic ubiquitin in synaptic plasticity and memory storage. *Cell* 147 1369–1383. 10.1016/j.cell.2011.09.056 22153079 PMC3442370

[B39] Qi Y. Hu N. W. Rowan M. J. (2013). Switching off LTP: mGlu and NMDA receptor-dependent novelty exploration-induced depotentiation in the rat hippocampus. *Cereb. Cortex* 23 932–939. 10.1093/cercor/bhs086 22490551

[B40] Ran I. Gkogkas C. G. Vasuta C. Tartas M. Khoutorsky A. Laplante I. (2013). Selective regulation of GluA subunit synthesis and AMPA receptor-mediated synaptic function and plasticity by the translation repressor 4E-BP2 in hippocampal pyramidal cells. *J. Neurosci.* 33 1872–1886. 10.1523/JNEUROSCI.3264-12.2013 23365227 PMC6619136

[B41] Richter J. D. Klann E. (2009). Making synaptic plasticity and memory last: mechanisms of translational regulation. *Genes Dev.* 23 1–11. 10.1101/gad.1735809 19136621

[B42] Richter J. D. Sonenberg N. (2005). Regulation of cap-dependent translation by eIF4E inhibitory proteins. *Nature* 433 477–480. 10.1038/nature03205 15690031

[B43] Roche K. W. O’Brien R. J. Mammen A. L. Bernhardt J. Huganir R. L. (1996). Characterization of multiple phosphorylation sites on the AMPA receptor GluR1 subunit. *Neuron* 16 1179–1188. 10.1016/S0896-6273(00)80144-0 8663994

[B44] Saba R. Storchel P. H. Aksoy-Aksel A. Kepura F. Lippi G. Plant T. D. (2012). Dopamine-regulated microRNA MiR-181a controls GluA2 surface expression in hippocampal neurons. *Mol. Cell. Biol.* 32 619–632. 10.1128/MCB.05896-11 22144581 PMC3266602

[B45] Selvakumar B. Jenkins M. A. Hussain N. K. Huganir R. L. Traynelis S. F. Snyder S. H. (2013). S-nitrosylation of AMPA receptor GluA1 regulates phosphorylation, single-channel conductance, and endocytosis. *Proc. Natl. Acad. Sci. U.S.A.* 110 1077–1082. 10.1073/pnas.1221295110 23277581 PMC3549090

[B46] Shehata M. Matsumura H. Okubo-Suzuki R. Ohkawa N. Inokuchi K. (2012). Neuronal stimulation induces autophagy in hippocampal neurons that is involved in AMPA receptor degradation after chemical long-term depression. *J. Neurosci.* 32 10413–10422. 10.1523/JNEUROSCI.4533-11.2012 22836274 PMC6703735

[B47] Silva A. J. Stevens C. F. Tonegawa S. Wang Y. (1992). Deficient hippocampal long-term potentiation in alpha-calcium-calmodulin kinase II mutant mice. *Science* 257 201–206. 10.1126/science.1378648 1378648

[B48] Swanger S. A. He Y. A. Richter J. D. Bassell G. J. (2013). Dendritic GluN2A synthesis mediates activity-induced NMDA receptor insertion. *J. Neurosci.* 33 8898–8908. 10.1523/JNEUROSCI.0289-13.2013 23678131 PMC3684268

[B49] Theis M. Si K. Kandel E. R. (2003). Two previously undescribed members of the mouse CPEB family of genes and their inducible expression in the principal cell layers of the hippocampus. *Proc. Natl. Acad. Sci. U.S.A.* 100 9602–9607. 10.1073/pnas.1133424100 12871996 PMC170964

[B50] Tsai L. Y. Chang Y. W. Lin P. Y. Chou H. J. Liu T. J. Lee P. T. (2013). CPEB4 knockout mice exhibit normal hippocampus-related synaptic plasticity and memory. *PLoS ONE* 8:e84978. 10.1371/journal.pone.0084978 PMC3875571 24386439

[B51] Udagawa T. Swanger S. A. Takeuchi K. Kim J. H. Nalavadi V. Shin J. (2012). Bidirectional control of mRNA translation and synaptic plasticity by the cytoplasmic polyadenylation complex. *Mol. Cell.* 47 253–266. 10.1016/j.molcel.2012.05.016 22727665 PMC3408552

[B52] Wagner J. J. Alger B. E. (1996). Homosynaptic LTD and depotentiation: do they differ in name only? *Hippocampus* 6 24–29. 10.1002/(SICI)1098-1063(1996)6:1<24::AID-HIPO5>3.0.CO;2-7 8878738

[B53] Wang C. F. Huang Y. S. (2012). Calpain 2 activated through *N*-methyl-D-aspartic acid receptor signaling cleaves CPEB3 and abrogates CPEB3-repressed translation in neurons. *Mol. Cell. Biol.* 32 3321–3332. 10.1128/MCB.00296-12 22711986 PMC3434545

[B54] Woo N. H. Nguyen P. V. (2003). Protein synthesis is required for synaptic immunity to depotentiation. *J. Neurosci.* 23 1125–1132 12598600 10.1523/JNEUROSCI.23-04-01125.2003 PMC6742257

[B55] Wu L. Wells D. Tay J. Mendis D. Abbott M. A. Barnitt A. (1998). CPEB-mediated cytoplasmic polyadenylation and the regulation of experience-dependent translation of α-CaMKII mRNA at synapses. *Neuron* 21 1129–1139. 10.1016/S0896-6273(00)80630-3 9856468

[B56] Xu L. Anwyl R. Rowan M. J. (1998). Spatial exploration induces a persistent reversal of long-term potentiation in rat hippocampus. *Nature* 394 891–894. 10.1038/29783 9732871

[B57] Yuen E. Y. Gu Z. Yan Z. (2007). Calpain regulation of AMPA receptor channels in cortical pyramidal neurons. *J. Physiol.* 580 241–254. 10.1113/jphysiol.2006.122754 17234699 PMC2075435

[B58] Zalfa F. Giorgi M. Primerano B. Moro A. Di Penta A. Reis S. (2003). The fragile X syndrome protein FMRP associates with *BC1* RNA and regulates the translation of specific mRNAs at synapses. *Cell* 112 317–327. 10.1016/S0092-8674(03)00079-5 12581522

[B59] Zhang M. Storm D. R. Wang H. (2011). Bidirectional synaptic plasticity and spatial memory flexibility require Ca^2+^-stimulated adenylyl cyclases. *J. Neurosci.* 31 10174–10183. 10.1523/JNEUROSCI.0009-11.2011 21752993 PMC3145492

[B60] Zhuo M. Zhang W. Son H. Mansuy I. Sobel R. A. Seidman J. (1999). A selective role of calcineurin Aα in synaptic depotentiation in hippocampus. *Proc. Natl. Acad. Sci. U.S.A.* 96 4650–4655. 10.1073/pnas.96.8.4650 10200317 PMC16387

